# 3D-Printing of Meso-structurally Ordered Carbon Fiber/Polymer Composites with Unprecedented Orthotropic Physical Properties

**DOI:** 10.1038/srep43401

**Published:** 2017-03-06

**Authors:** James P. Lewicki, Jennifer N. Rodriguez, Cheng Zhu, Marcus A. Worsley, Amanda S. Wu, Yuliya Kanarska, John D. Horn, Eric B. Duoss, Jason M. Ortega, William Elmer, Ryan Hensleigh, Ryan A. Fellini, Michael J. King

**Affiliations:** 1 Lawrence Livermore National Laboratory, 7000 East Ave., Livermore, CA94550, USA

## Abstract

Here we report the first example of a class of additively manufactured carbon fiber reinforced composite (AMCFRC) materials which have been achieved through the use of a latent thermal cured aromatic thermoset resin system, through an adaptation of direct ink writing (DIW) 3D-printing technology. We have developed a means of printing high performance thermoset carbon fiber composites, which allow the fiber component of a resin and carbon fiber fluid to be aligned in three dimensions via controlled micro-extrusion and subsequently cured into complex geometries. Characterization of our composite systems clearly show that we achieved a high order of fiber alignment within the composite microstructure, which in turn allows these materials to outperform equivalently filled randomly oriented carbon fiber and polymer composites. Furthermore, our AM carbon fiber composite systems exhibit highly orthotropic mechanical and electrical responses as a direct result of the alignment of carbon fiber bundles in the microscale which we predict will ultimately lead to the design of truly tailorable carbon fiber/polymer hybrid materials having locally programmable complex electrical, thermal and mechanical response.

Carbon fiber (CF)/polymer composites are a transformative class of high-performance, lightweight material, where high aspect ratio carbon fibers^1^,^2^,^3^,^4^,^5^ reinforce a polymer matrix (i.e., a highly crosslinked, aromatic thermoset resin) to enhance mechanical, electrical, and thermal characteristics^6^,^7^. They have found wide application in the aerospace^8^, automotive^9^, civil^10^, and energy storage^11^ areas due to their high strength-to-weight ratio^12^,^13^, high specific properties^14^ and potential multifunctionality^15^. However, conventional manufacturing processes significantly hinder further development and broader application of these materials. For example, high performance continuous fiber composites can be conventionally fabricated by several processes, including hand lay-up^16^, filament winding processes^17^ or vacuum assisted resin transfer molding^18^. However these methods are typically costly, often only viable for a limited subset of part and fiber pattern geometries, and can also lack reliability and repeatability in manufacturing - due to limited control over the composite mesostructure^19^. In contrast, short or chopped fiber composites have become practical and low-cost alternatives with comparably improved physicochemical properties^20^,^21^,^22^. In comparison to the wound composites, short carbon fiber composites can be fabricated by extrusion compounding or injection molding processes in a relatively facile manner^23^,^24^,^25^,^26^. It is noteworthy that the mechanical properties of these composites largely depend on the fiber length, orientation and distribution^20^,^22^,^27^,^28^, as well as fiber-matrix interfacial adhesion^1^,^29^. During the manufacturing process, fiber breakage occurs^20^ due to the fiber-fiber^23^,^30^ and fiber-polymer^20^ interactions, attenuating the mechanical properties of the final composite part^31^,^32^. Similarly, directionality of fiber orientation is also affected by the aforementioned factors. Therefore, the processing method, matrix material, and fiber loading will determine the ultimate performance of both continuous and short fiber composites and can be further altered by tuning their mesostructure and geometric design.

3D printing is a category of sequential deposition methods allowing the creation of complex structures with controlled composition, geometric shape, and functionality that is unattainable through traditional manufacturing techniques^33^,^34^. Current success in 3D printing of fiber composites relies on the fused deposition modeling (FDM) of mechanically weak, thermally unstable thermoplastic fiber reinforced feed stocks^35^,^36^,^37^,^38^,^39^. Recently, a filament-based, ambient temperature 3D printing technique, known as direct-ink writing (DIW) has been utilized to fabricate fiber (i.e., carbon nanotube, ceramic)-epoxy composites^40^,^41^. DIW is a high-speed and low-cost 3D printing technique that employs a multi-axis motion stage to assemble complex 3D structures by robotically extruding continuous “ink” filaments through the micro-nozzle in a layer-by-layer scheme^42^,^43^,^44^. Ideal ink materials used in DIW should possess shear-thinning behavior with a moderate yield stress to facilitate extrusion flow under shearing force and a quick pseudoplastic to dilatant recovery with excellent shape retention after deposition to maintain the printed geometry^45^,^46^,^47^. This method has been used previously to fabricate ceramic and polymeric scaffolds^48^,^49^, antenna and sensors^40^,^50^,^51^, microbatteries^52^, optical waveguides^53^, and graphene aerogels^54^ with feature sizes ranging from microns to millimeters in size. However, there are still few examples that fully leverage the potential of 3D printing technologies to create carbon fiber composite architectures using thermosets as structural matrices^55^,^56^. Especially, a major challenge in realizing 3D printing of high performance aerospace-grade carbon fiber and polymer composites lies in the utilization of high strength, high glass transition temperature (T_g_) thermosetting resins as the polymer phase. Current commercial aerospace resins are typically amine cured, two-component aromatic epoxide systems. These systems have the desired physical properties, but require extensive time periods of multiple hours to days in order to achieve a full cure, have limited rheological profiles and pot-life. Therefore, due to the slow cure profiles of amine/epoxy feed stocks, they are not suitable for use in continuous lay-down or printing process where the resin component plays an immediate and active role in orienting and constraining the fibers’ direction and alignment. This limitation also prevents the construction of self-supporting structures of any complexity on a reasonable manufacturing timeframe (minutes/seconds).

Here, we report a novel thermoset resin system specifically tailored for DIW process with curing characteristics (gelation in 1–5 seconds, full density cure in 10 minutes) and rheological properties (shear thinning and viscosity matched with the CF phase) that allows the printing of complex 3D structures in real time, yet has final mechanical properties that meet or exceed current commercial amine cured epoxy resins. We demonstrate that our approach allows the formation of 3D micro-structurally architected CF/polymer composites with enhanced physical properties. Characterization of these architected materials has demonstrated that we have unprecedented control of fiber orientation and alignment and it is that control of composite mesostructure, which translates to the improved, and novel physical responses we observe and characterize in these materials. Furthermore, we predict that this enhanced control of the composite mesostructure will provide the opportunity to use advanced design optimization techniques to achieve greatly enhanced macroscopic performance and predictability in physical response.

## Results and Discussion

### 3D-Printing of shear-aligned short fiber composites

#### Filamentary extrusion of carbon fiber/resin inks to form freestanding pre-AMCFRC structures

**Figure 1:**
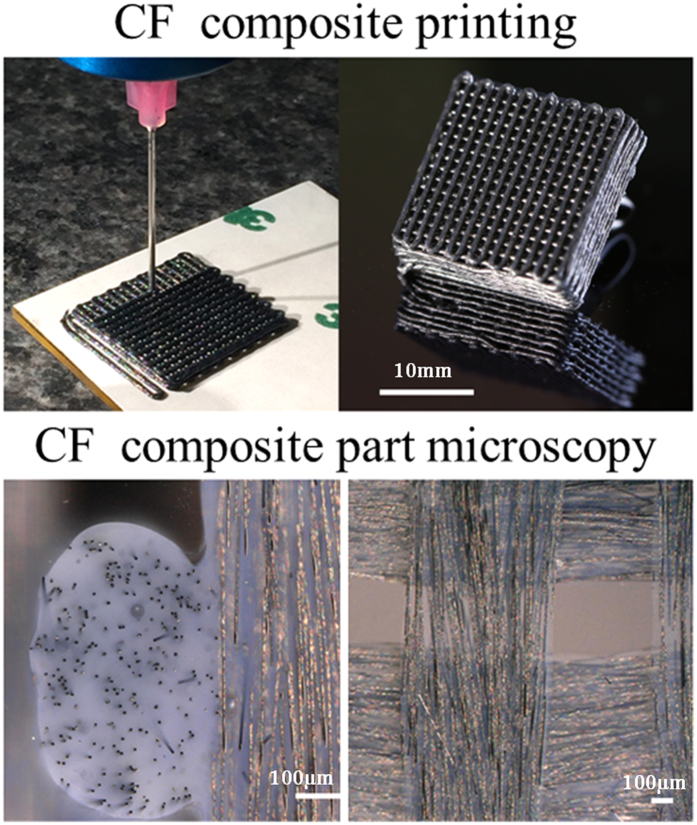
Top left, microextrusion of an 8% volume fraction carbon fiber loaded bisphenol-F resin ink from a 600 μm straight tip nozzle under a constant driving pressure. The average fiber thickness and lengths were 6 and 300 μm respectively. Note that the carbon fiber loaded ink will flow as a contiguous fluid under pressure at measured volumetric flow rates of 7.4 × 10^−4^ cm^3^ min due to the shear thinning behavior of the resin phase and the efficient shear alignment of the high aspect ratio fiber phase during printing. The ink rapidly solidifies on extrusion to form self-supporting filaments on the size scale of the extrusion nozzle tip (top right) which may be built up from a base substrate into 3D structures, shown here is an open cell 0, 90 arrangement with an average filament diameter of 600 μm and that was subsequently thermally cured to yield a robust CF composite with a nominal 50% porosity. (Lower) micrographs of y-z plane (left) and x-y plane (right) sections of the printed and cured AMCRFC part. It can be noted from both images that we achieve both a high degree of control of the micro-architecture of the part and the relative alignment of the fiber-phase within individual filamentary extrudates.

Direct ink writing (DIW) 3D printing processes have been shown by the authors and others to be a highly effective means of creating ordered structures with tailored and predictable properties^44^,^54^,^57^. DIW offers exquisite control over the structural arrangement of material and void, which enables the formation of 3D structures with controlled or architected microstructures. The DIW process relies on the controlled extrusion of the desired material (ink) from a micro nozzle onto a substrate which is translated in the x, y and z planes to form a 3D structure made up of a given arrangement of filaments of the ink. The ability to form an ordered, self-supporting structure in real time is dictated by the rheological properties of the ink which - must act as a fluid under the force of extrusion, yet rapidly solidify, through either a physical or chemical process on a timescale commensurate with the printing process. In this work, we have based our ink on a bisphenol-F epoxy resin oligomer (BPFE) system which we have modified with both colloidal silica and dispersed high aspect ratio, discrete carbon fibers. This carbon fiber loaded ink has been shown to be extrudable at room temperature through nozzles with minimum exit diameters of 250 μm with rapid physical solidification into a filamentary extrudate and displays the rheological characteristics necessary for a processable DIW substrate (Fig. 1).

From Fig. 1 it can be observed that our carbon fiber loaded ink, at demonstrated CF loadings of 8 volume%, is capable of being extruded into high resolution structures with individual filamentary diameters of ~600 um (AMCFRC’s with filamentary diameters ≥250 μm have also been demonstrated, examples of which may be found in the supporting information section, Figure S1) and that are self-supporting prior to the initiation of crosslinking reactions to cure the thermoset epoxy resin phase and form a final AMCFRC structure. It can be observed from the microscopy data presented in Fig. 1 that through our AM process we are able to both A) significantly align bundles of carbon fibers within a polymer matrix by means of shear alignment. B) Accurately place those aligned bundles of fibers in the x, y, z plane in effect to make a virtual layup of fiber bundles in an analogy of a bulk continuous fiber weave layup process.

**Figure 2:**
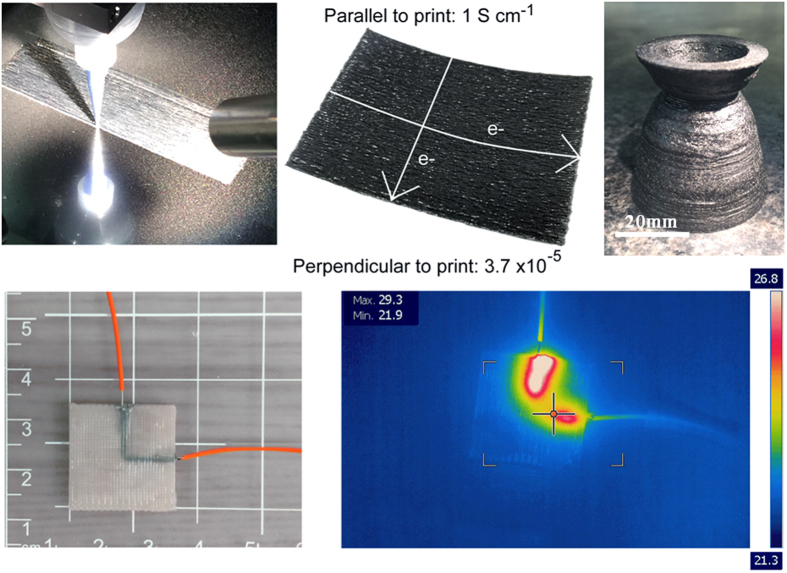
Upper left, a single layer uniaxially printed AMCRFC. Upper middle, the single layered part exhibiting a designed orthotropic electrical response where measured electrical conductivities (4 point probe) parallel to the fiber direction are in the order of 1 S cm^−1^ whereas the conductivity in the direction perpendicular to the fiber alignment direction is ~5 orders of magnitude lower. Upper right, a fully 3D parabolic nozzle structure AMCRFC part. Lower left – a basic example of a thermally conductive carbon fiber rich resin region printed in fiber poor thermoset matrix. Lower right – thermal image of the conductive channel as it is resitively heated by the applicaiton of a d.c. current, representing the proof of concept of a conductive thermal path printed via a 3D-printing process.

The use of high volume fractions (>20 volume%) of high aspect ratio (l/d > 50) carbon fibers is necessary for the formation of high performance composites^58^, presents a challenge for any DIW 3D printing process that wishes to retain a high degree of feature resolution (<1000 μm) and some control over the orientation of fibers in a given feature. It is therefore both desirable and necessary to control the fiber registration and orientation within the matrix and maintain the fluid ink properties required for processing. For the purposes of these preliminary investigations we have fixed an upper carbon fiber loading limit of 8 volume % in the pre-crosslinked ink, however with more advanced nozzle geometries it is feasible that the absolute loading level of fibers in the final extrudate may be increased beyond this value to approach volume fractions ≥20 volume %. Critical to gaining an advantage over conventional short fiber carbon/polymer composites is the ability to align the fibers in a given orientation and at a given resolution. In DIW, like any extrusion process, there is a significant driving force for particle alignment in the direction of flow^59^. However, in DIW microextrusion coupled with the ability to write with 10’s μm accuracy in the x, y, z planes offers the potential to spatiality orient high resolution bundles of particles in a given 3D build space arbitrarily, yielding new degrees of freedom in composite micro-architecture. The practical realization of this process in a carbon fiber/polymer composite ultimately depends on the ability to continuously align high aspect ratio fibers within a matrix and extrude reliably, without the loss of filamentary resolution or clogging of the print head. We observe experimentally that the fiber phase of our carbon fiber loaded ink initially consists of randomly oriented, dispersed carbon fibers on the order of 600 μm in length. On microextrusion through the DIW print head the fiber phase is however forced to undergo shear alignment. This effect was reported by both Lewis^41^ and Tekinalp^55^, but at significantly lower aspect and fiber volume fractions in the DIW based process demonstrated by Lewis. And while Tekinalp achieved fiber alignment at significant volume fractions of up to 25% volume CF by volume using an fused deposition modeling (FDM) approach, this was exclusively in an acrylonitrile butadiene styrene (ABS) *thermoplastic* matrix which while easy to process, does not have the ultimate mechanical or thermal properties suitable for the production of high performance aerospace composite applications - where aromatic thermoset resins are exclusively employed. We therefore believe that this is the first demonstration of the successful microextrusion of a high aspect ratio fiber filled aromatic thermoset resin system at volume fractions above 1% v/v using DIW. See Fig. 2 and Video S1 in the supporting data section.

From Fig. 2 it may be observed that unlike traditional macroscale CF composite processes, we are not limited to simple ordered arrangements and it is possible to print a wide variety of structure, geometries and patterns of carbon fiber composite material using this technique including in this example a di-parabolic rocket nozzle form. Through the use of additional resin feeds it is possible to vary the loading of carbon fibers in a given area of the build and through controlling local orientation it is possible to introduce a high degree of orthotropy into the physical properties of the final AMCFRC structure. Indeed we have demonstrated here, both the ability to effectively introduce and control both orthotropy in electrical conductivity, thermal channeling within a structure as well as controlled orthotropy in mechanical response.

We have demonstrated that such continuous control of carbon fiber orientation and loading within a build space opens the door to greater control and optimization of physical properties and even novel functionality within a material. However, it must also be stated that to a very large extent the properties and performance of such additively manufactured composite materials remains heavily dependent on the chemical structure and physical morphology of the polymer network used as the resin phase, therefore both understanding and controlling the structure and properties of the resin are essential to the development of robust AMCFRC fabrication strategies.

### Rheological characterization and modeling of AMCFRC forming carbon fiber/resin inks

**Figure 3:**
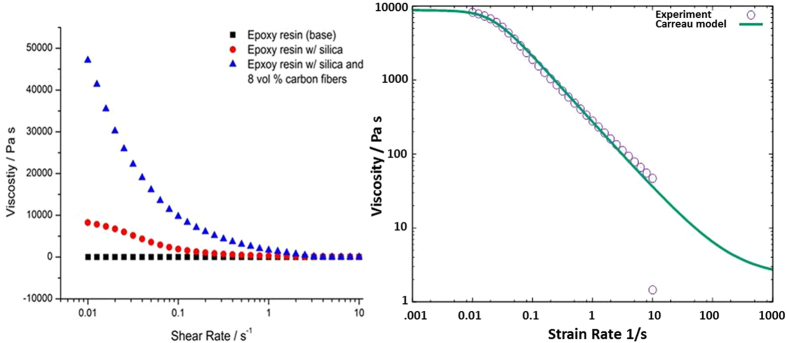
Left, experimentally derived shear rate dependent viscosity profiles at 30 °C for the unmodified bisphenol-F oligomer resin, the resin modified with 4 volume % of high surface area pyrogenic silica nanoparticles and a complete ink formulation consisting of the resin, silica and 8 volume% 600 μm length carbon fibers. Note that while the base resin behaves as a Newtonian fluid, the addition of the silica nanoparticles yield a non-Newtonian system with a thixotropic shear dependent response that matches closely a Carreau fluid model (right). The addition of carbon fibers to this fluid matrix increases the static viscosity by ~ four times however the fully CF loaded ink still exhibits a significant and useful shear thinning response, albeit at higher effective shear rates.

As stated previously, the ability for a given polymer ink formulation to be ‘writable’ using a DIW process is in a large part, dependent on the static and flow rheology of the formulation. The rheological properties of the CF/epoxy ink used in the manufacture of our AMCRFC materials are therefore a consequence of the physical multiphase structure of the ink (Fig. 3).

From the rheological characterization of the CF ink formulation in Fig. 3 it can be observed that the oligomeric, Newtonian BPFE resin is modified to behave as a thixotropic, non-Newtonian fluid by the addition of a low volume fraction of high surface area silica nanopartices. The shear rate dependent response of this silica modified resin has been shown to conform to a classical Carreau fluid model, of the form given in Eq. 1.


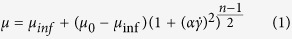


where μ_0_ = the viscosity at a shear rate of 0, determined to be ~8000 Pa s, μ_inf_ = viscosity at an infinite shear rate, which is chosen to be 2.2 Pa s, 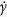 is a shear rate and where the coefficients a, n, were determined to be 45 and 0.1 respectively for the silica modified BPFE resin (see Fig. 3).

Significantly, it has been observed experimentally that if carbon fibers are dispersed directly into the unmodified Newtonian resin, the resultant system will not extrude from a DIW nozzle under pressure. The resin and fibers will in fact phase separate, with the resin flowing past a static fiber phase in the barrel/nozzle assembly. It is hypothesized that the higher viscosity silica modified resin induces significantly increased drag forces between the fiber and the resin phases and allows the fiber phase to be effectively ‘carried’ as a contiguous component of the fluid matrix.

It is clear from these data shown in Fig. 3 that despite the increases in ‘static’ viscosity (as shear rate → 0) that are observed on the addition of carbon fibers to the silica modified resin, the ink still shows a clear thixotropic response and reaches a minimum viscosity of ~2 × 10^3^ Pa s - which approaches that of the silica modified resin under shear, at rates ~2 reciprocal seconds. This observation suggests that as the shearing forces develop in the extrusion nozzle, the ink will effectively flow despite its high loading of fibers. The comparatively high static shear viscosities (~5 × 10^4^ Pa s) support the observation that the fluid rapidly relaxes, physically solidifies rapidly on removal of the shearing force at the point of extrusion and we have demonstrated that it is possible to print self-supporting structures from this ink formulation that maintain individual fibular feature resolutions on the order of 250 μm.

Gaining an understanding beyond simple bulk rheology, of the fluid dynamics of the extrusion process with this complex, multi-phase system are ultimately important for the further development and implementation of such DIW methods for the manufacture of AMCFRC materials. And we are using computational modeling to better understand flow behavior and fiber alignment during manufacturing process.

**Figure 4:**
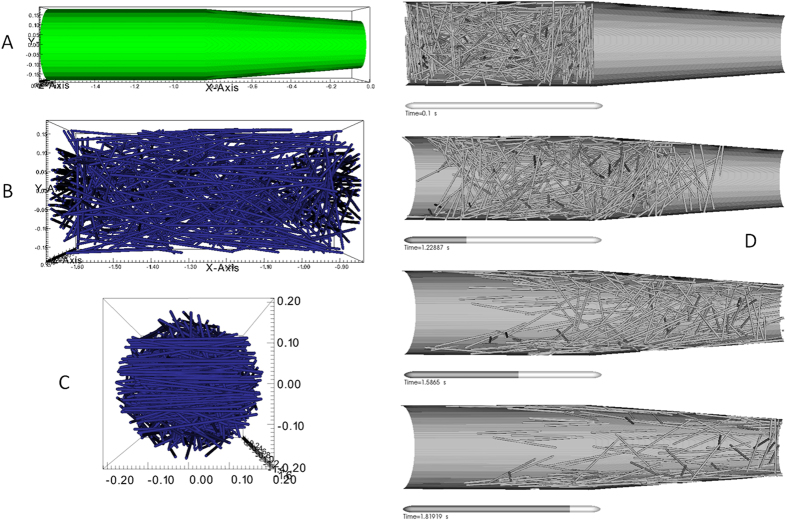
High resolution numerical simulation of a carbon fiber loaded ink under conditions of micro-extrusion during a DIW printing process to form an AMCFRC. (**A**) Represents the total computational domain for these simulations which cylindrical/conical volume, terminating in a 400 μm orifice and is representative of the last 2 mm of the actual extrusion head. (**B** and **C**) are side and end views showing the initial (random) fiber partial distributions within the contiguous phase. (**D**) represents time the resolved evolution of the fiber orientations in 3D within the computational domain under simulated conditions of printing. Note that the simulation predicts what appears to be wall dominated shear alignment process which follows closely the predicted velocity profile of the fluid.

Using a numerical model described in detail in the experimental section, we have been able to simulate at high resolution, short fiber flow at a volume fraction of 8% of the total ink volume, matching current ink loadings. The fiber dimensions simulated were 6 um diameter and 300 um length. The rheological behavior of the silica modified resin was simulated as a non-Newtonian fluid with a viscosity that is dependent on the shear rate as shown in Fig. 3 and described in the previous section. The silica particles are simulated as a continuum medium however the carbon fibers are allowed to interact with each other and the walls in the simulation and are modeled as discrete particles. The nozzle extrusion diameter simulated was 400 um. The nozzle has a conical shape with an upper cylindrical internal diameter of 5 mm and a long axis length of 31 mm terminating in a 400 um cylindrical tip (Fig. 4).

Since the exact position of the fibers in the nozzle is unknown, a random fiber distribution was generated inside the computational domain using the ALE3D ParticlePack algorithm^60^. The initial distribution of fibers having a volume of fraction of 8% in the cylindrical section is shown in Fig. 4(B,C). Preliminary results of fiber evolution in the nozzle as a function of extrusion time at constant pressure are shown in Fig. 4(D). It can be observed that as the simulation develops within the computational domain, the fibers do indeed align preferentially in the direction of extrusion. At early times fiber alignment occurs mainly at the walls where the shear forces are greatest however as the simulation proceeds it is apparent that the alignment of fibers proceeds into the bulk of the fluid domain, mirroring the parabolic velocity profile of the contiguous fluid. These simulations strongly suggest therefore that it is not only the internal surface to volume ratio of the nozzle that is important in inducing shear alignment but that the rheological properties of contiguous phase fluid are also an important factor in achieving efficient alignment during extrusion. These simulation results both validate and explain what was observed experimentally – that to be an efficient ‘carrier’ for the carbon fiber phase, the pre-polymer resin must present sufficient viscous drag forces in the fibers so as to induce both bulk flow, separation and alignment of the carbon fiber phase under shear.

**Figure 5:**
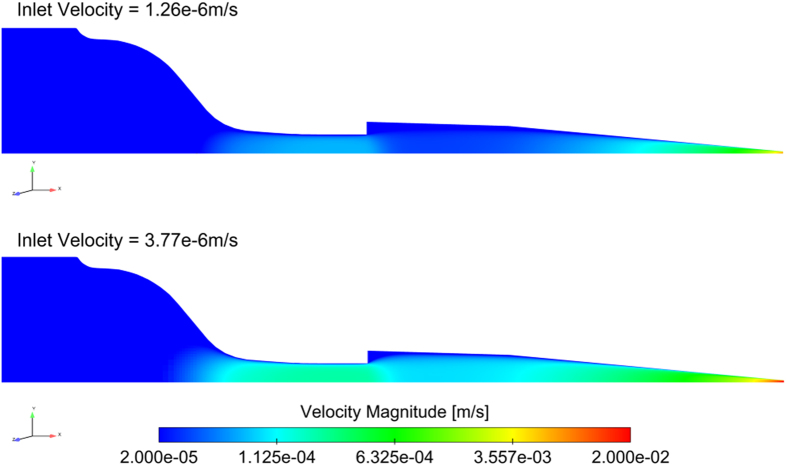
CFD simulations of a CF filled epoxy ink flow through a standard 10cc DIW syringe body and 250 μm cylindrical extrusion tip at 2 simulated print speeds, 5 mm/min extrusion (top) and 15 mm/min (bottom), with an inlet velocity selected such that the outlet (average) velocity matches the head speed. The viscosity model which the fluid is based on is derived from the experimental rheology data obtained for the 8 vol.% AMCFRC ink.

High resolution numerical simulations are effective in modeling aspects of the DIW printing process for AMCFRC materials. However, the modeling technique is currently in a developmental form, is time consuming and computationally expensive. Thus, simulations are limited to only a small fraction of the DIW extrusion process. In order to obtain a global flow perspective of what is occurring within the printing system, we simulated the extrusion process on a larger size scale. For this broader view of fluid dynamics, we employed a secondary approach, which utilizes a commercial computational fluid dynamics (CFD) code (STARCCM+), to model the bulk flow within various DIW print head geometries. While this approach does not explicitly model discrete fibers, it allows for the global flow dynamics of a given print head geometry to be assessed effectively. Shown in Fig. 5 are the results of CFD generated simulations of the micro-extrusion process of an 8 volume % CF loaded epoxy ink.

As the ink is pushed from the syringe barrel into the nozzle, it enters a constriction causing the bulk velocity to increase. The cross section then abruptly increases in diameter which results in rapid deceleration of the fluid. While the high shear rates in the constriction may promote shear alignment of the fibers in the axial direction, these fibers may be compressed as they enter the expansion. This could lead to clumping or clogging within the nozzles. To reduce this potential complication, future nozzle geometries will need to be considered. In particular, it may be beneficial to design a nozzle characterized by a monotonic decrease in diameter as the ink moves from the syringe barrel to the nozzle tip.

### Tailored chemical crosslinking to form fully dense composite architectures

#### Network chemistry and cure characterization

**Figure 6:**
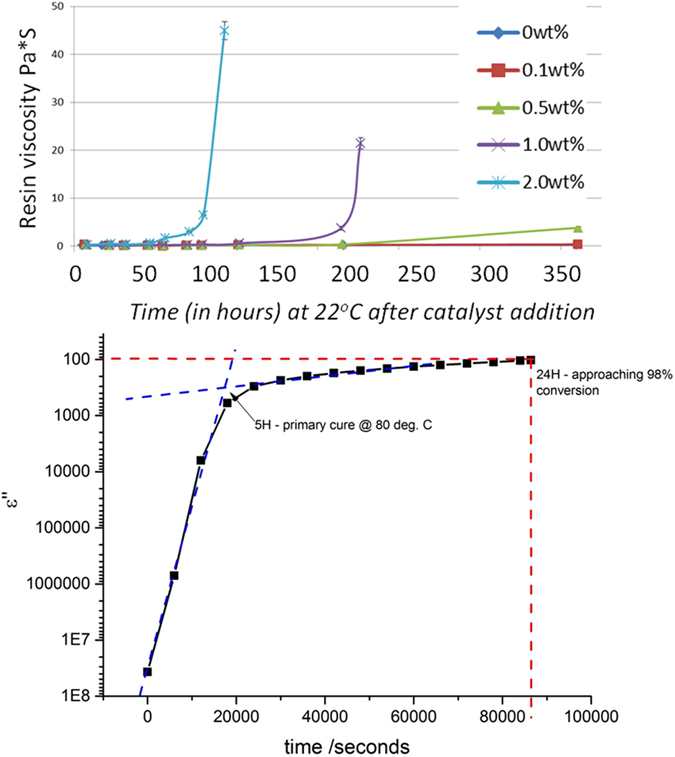
Rheology and dielectric relaxometry characterization of the curing performance of the homopolymerized epoxy ink system as a function of catalyst loading and temperature. Top panel: Room parallel plate oscillatory strain rheology measurements of the BPF resin on which we base our ink, at catalyst loadings of 0.1–2 wt% (conducted at room temperature. Note that at high catalyst loadings, the effective work time of the resin is ~75 hours and at low loadings (0.1 wt%) the room temperature work time is >350 hours. Lower panel: dielectric relaxometry analysis of the effective network curing process in the based BPF resin, at a catalyst loading of 0.1 Wt%. Despite being stable for over 250 hours at this low loading, these data show that a primary ‘green strength cure’ may be attained in only 5 hours at 80 °C, with full conversion of ~24 hours. At higher temperatures (150–200 °C) thermal snap curing may be achieved in under a minute. Such rapid curing is difficult to assess using relaxometry, but may be observed qualitatively in Video S2.

In this work we have employed latent thermal cure catalysis, which through the generation of a strong Lewis acid above ~70 °C, can efficiently ring open oxriane groups and therefore initiate a thermally activated self-crosslinking, homopolymerization reaction between the epoxide functionalities of the BPF resin. Latent cure catalysis via strong Lewis acid generators (such as Boron trifluoride salts^61^) yield a route towards epoxide homopolymerization which is an attractive alternative to conventional two component amine cured thermoset epoxy resin systems for DIW applications. Controlled homopolymerization eliminates the necessity to combine the reactive polymer components immediately prior to or during the printing process. In addition, our resin ink system requires only moderate post-curing stage to achieve full network density (80 °C for 12 hours) at low catalyst loadings (0.1 Wt%). Furthermore, due to its advantageous temperature and reactivity profile, which enables long resin lifetimes at ambient temperatures, while maintaining a rapid curing response at elevated temperatures, formulated inks may be printed on a flexible timescale yet rapidly cured when required for the formation of complex structures. Shown in Fig. 6 are the results of rheological and dielectric relaxometry studies of the curing response as a function of temperature and catalyst loading for a series of formulated AMCFRC resin inks.

**Figure 7:**
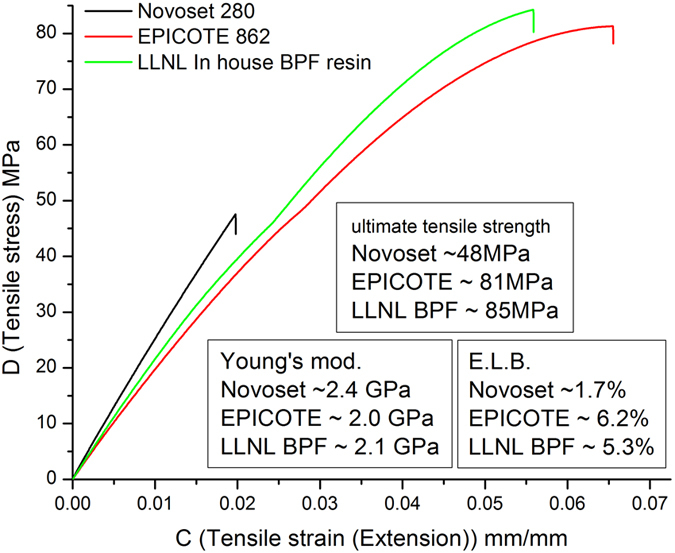
Mechanical properties of the BPF homopolymerized, latent cured resin vs. commercially available high performance thermosets. Tensile testing of cured specimens of our in-house BPF resin system were compared to both a conventional amine cured BPF (Epicote 862, cured with DETDA - as per the manufacturers guidelines) and Novoset 280 Cyanate ester resin (cured with Cu (ACAC)_2_ per the manufacturers guidelines). Note that our resin system has an increased Young’s modulus, comparable ultimate tensile strength and decreased elongation at break compared to that of the commercial BPF – it is in effect, stiffer and stronger. And while it is less stiff than the cyanate ester resin, it is significantly less brittle.

From these data it can be observed that while the rheological properties of the formulated resin can remain stable at 20 °C for over 5 days at catalyst concentrations below 2 wt%, gelation of the system and curing to full network density can be achieved in less than 5 seconds for a given temperature/catalyst loading profile if so desired. Such thermal snap curing, attainable at temperatures of 150–200 °C is difficult to assess using relaxometry, but may be observed qualitatively in Video S2. Mechanical analysis of these homopolymerized networks (Fig. 7) demonstrate clearly that their mechanical performance compare favorably with that of conventional amine cured BPF systems, aromatic cyanate ester resins and demonstrates that controlled homopolymerizaiton may be employed to obtain high quality thermoset network resins.

#### Final properties of 3D printed AMCFRC materials

**Figure 8:**
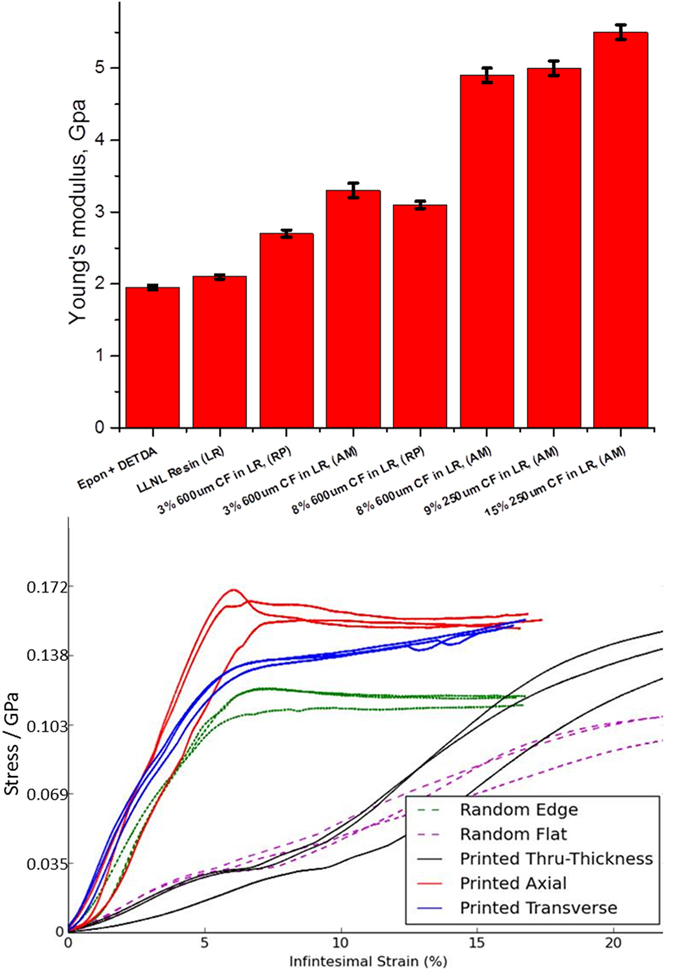
Tensile and compressive testing results from AMCFRC and equivalent vol. fraction random pressed parts. Upper: Average Young’s modulus as determined by tensile testing for unfilled resins, AMCFRC samples and pressed chopped fiber parts. Two fiber lengths (250 and 600 μm) and 4 volume fractions of fiber (0, 3, 8 & 15%) were investigated RP = random pressed and AM = additively manufactured respectively. In these parts, the preferential direction of fiber alignment will be in line with a single axis, the major axis of printing -referred to as the ‘print direction’ throughout the printed part. This is also illustrated in Fig. 9. Note that the AMCFRC parts outperform random chopped fiber parts even at lower volume fractions of fiber. Lower: compressive testing of 3 vol.% AMCFRC and random parts. Note that AMCFRC parts outperform random chopped parts not only when they are tested axially to the print direction but in both transverse and through thickness modes as well.

The mechanical properties of our 3D printed AMCFRC systems have been assessed in both tensile and compressive modes as a function of CF volume fraction and fiber length compared with both in-house and commercial unfilled BPF reins, and chopped CF filled random, pressed parts. All AMCFRC parts were printed unidirectionally and tensile tested in the print axially to the print direction, all AMCFRC parts were compression tested both transverse, axially to the print direction and through thickness. Shown in Fig. 8 are the results of both the tensile and compressive testing.

It can be observed from the tensile data that our AMCFRC 4 layer unidirectioally printed parts in the axial direction, significantly outperform their random, pressed counterparts even at lower CF volume fractions. For example, a 3 vol % 610 μm AMCFRC exhibits a 6% *increase* in Young’s modulus versus a 8 volume % random pressed part and an 8 vol.% AMCFRC is has a Young’s modulus that is 37% higher than the equivalent volume fraction random pressed part. We attribute this significant increase in modulus to volume fraction entirely to the preferential CF alignment gained through the DIW process. And in effect, the AMCFRC parts exhibit a large othrotropic response: It may be estimated that in a conventionally molded chopped or discontinuous fiber part, with a random distribution of fibers throughout the volume that for any given stress direction, only ~1/3 of the fibers are aligned in axially to the applied stress. In our highly aligned fiber AMCFRC systems we estimate that 80–90% of the fibers are aligned in the desired axis. Therefore the number of fibers that may effectively contribute to the stress distribution for a tensile case is greatly increased per unit volume. These tensile data in effect validate the concept that if discontinuous fibers can be effectively aligned throughout the volume of a composite part, then the mechanical performance of that part may be greatly improved for a given volume fraction of fiber.

If the compressive case is examined, we gain further insight into the benefits of AMCFRC’s obtained through the DIW process versus conventional molded fiber composites. In these data given in Fig. 8, 600 μm 3 vol.% conventional random pressed CF parts are compared to equivalent 4 layer unidirectionally printed AMCRFC’s in compressive performance both through thickness and edge on. Furthermore, we have tested the AMCFRC’s not only through thickness and edge on axially to the print direction, but also transverse to the print direction. From these data given in Fig. 8, it is immediately apparently that the AMCFRC’s tested on edge axially; outperform a random pressed part (edge on) in compression. However we also observe that the AMCFRC’s tested through thickness and edge on *transverse* to the print direction also outperform random pressed parts in compression. These results are somewhat counter intuitive initially as one may assume that if we align in one direction (Axial), to maximize performance then we lose in another (transverse). However it is in fact that case that a regular, aligned filamentary array of fibers will in fact have an improved compressive response to a purely random distribution within a volume.

**Figure 9:**
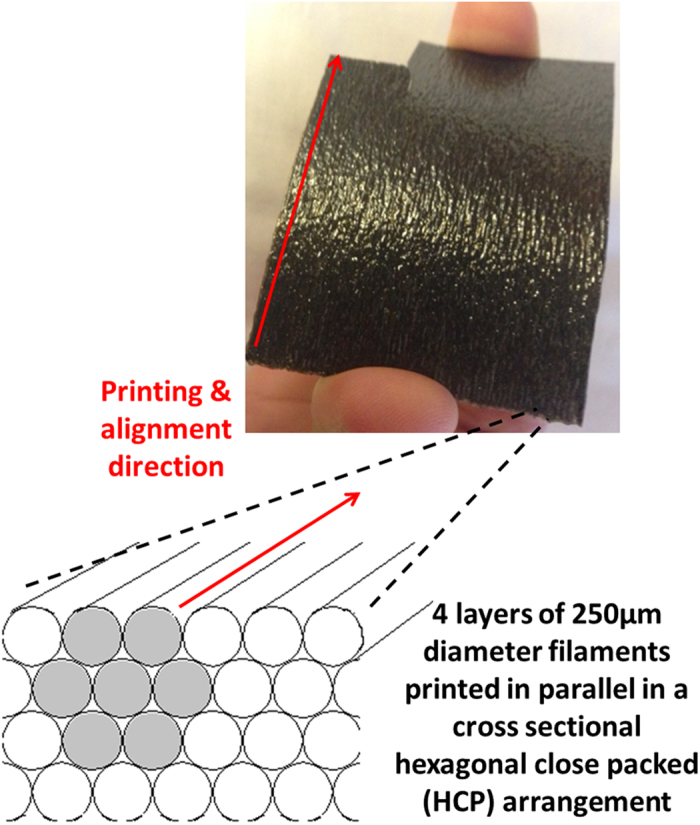
Illustration of a uniaxially printed AMCFRC part with 3 vol.% carbon fiber, of average length and diameter of 300 and 6 μm respectively. Note that this is a 4 layer printed part where each individual printed filament lies parallel to each other in a hexagonal close packed (HCP) arrangement - with the cnsequence that the preferential direction of fiber alignment will be in line with a single axis throughout the printed part, referred to as the printing direction.

In addition to assessing the orthotropy in mechanical properties, we have assessed the electrical response of a subset of AMCFRC materials. A 3 volume % CF loading AMCFRC resin was printed in a uniaxial layup at a filamentary print resolution of 250 μm in a 4 layer, hexagonal close packed pattern. An illustration of this part is given in Fig. 9.

A series of four point probe surface electrical conductivity measurements were made on the printed part shown in Fig. 9, with electrode arrangements both parallel and perpendicular to the printing (and fiber alignment) direction within the part. In measurements taken *parallel* to the printing direction, the average surface conductivity was determined to be an average conductivity of 1.1 S cm^−1^. Whereas the conductivity measured perpendicular to the printing direction was consistently determined to be orders of magnitude lower with an average conductivity of 3.7 × 10^−5^ S cm^−1^. This large directional disparity in surface conductivity may again be explained by the highly orthotropic nature of the AMCFRC parts. In printing and aligning in a uniaxial manner we have been able to impose a large degree of directionality on the electrical response of a printed part. From this basic demonstration it is however clear that more complex multi-dimensional control of electrical properties within such AMCFRC parts is possible.

## Conclusions

In this work, we have reported a significant advance in the scientific and technological development of micro-extrusion 3D printing techniques for the additive manufacture of high performance, high aspect ratio carbon fiber filled thermoset composite materials. This development has been enabled by the use of versatile, new homopolymerized epoxide based resin chemistries and the large degree of microstructural control afforded by Direct Ink Writing (DIW) technology. We reported the highest volume fraction and aspect ratio of carbon fibers extruded in an aromatic thermoset ink to date. Furthermore, we have demonstrated the utility of modified DIW techniques for efficient shear alignment of carbon fibers during a printing process. Through computational modeling and simulation we may now predict and optimize this process to maximize the degree of fiber alignment within an extruded filament. Additionally, we have clearly demonstrated that with the microstructural control and degree of alignment afforded by DIW, it is possible to fabricate AM composites which outperform equivalent volume fraction random pressed parts in mechanical response and which exhibit controlled and tunable orthotropic electrical and mechanical response.

## Experimental Section

### Materials

Oligomerized Bisphenol-F diglycidyl ether (BPF) and Diethyltoluenediamine (DETDA) were obtained from Hexion (USA). Novoset 280 Cyanate ester resin was generously donated by Novoset LLC (US). All carbon fiber used in this study was Grade HTS40 obtained from Toho Tenax (Japan), having a fiber tensile modulus of 240 Gpa, 1.8% elongation at break and a mean density of 1.77 gcm^−1^. Cabosil TS530 fumed silica was obtained from Cabot inc. (USA). All other reagents and materials used were obtained from Sigma Aldrich (US).

### Preparation of AMCFRC Inks

10 g of partially oligomerized Bisphenol-F diglycidyl ether F with 0.1 Wt% catalyst was massed into a polypropylene mixing cup (FlacTec inc) and to this 15 Wt% of Cabosil TS-530 silica were added. The resin/silica combination was mixed in an off-axis centrifugal mixer (FlacTec inc.) at 2500 rpm for 1 minute, followed by vacuum degassing at room temperature in a vacuum oven (Cascade Tec. Inc) for 20 minutes. 3–15 Volume % of chopped carbon fiber was added to the degassed mixture and mixed at 2500 rpm for a further 1 minute prior to a repeat degas step for 12 hours at room temperature.

### Direct Ink Writing of AMCFRC structures

Prepared AMCRRC inks were loaded into a 3 ml syringe barrel (EFD) attached by a luer-lok to either a smooth-flow tapered nozzle (250, 400 or 610 μm inner diameter) or a straight tipped 600 μm exit diameter, 2 cm long stainless steel nozzle for the preparation of the 8% by volume AMCFRC materials shown in Fig. 1 and used in all mechanical analysis. An air-powered fluid dispenser (Ultimus V, EFD) provided the requisite pressure to extrude the ink through the nozzle. The target patterns were printed using an x-y-z 3-axis positioning stage (ABL 9000, Aerotech). The 3D structures were printed onto fluoropolymer-coated glass slides, with a nozzle height of ~0.75 times the nozzle diameter to ensure moderate adhesion to the substrate and between printed layers. Complete, printed structures were transferred including the substrate to a forced convection oven (Binder) where they were cured at 80 °C for a period of 12 hours prior to removal from the substrate and a 24 hour post cure at 120 °C.

### Numerical modeling

The numerical model we used to simulate the chopped fiber flow is based on the Distributed Lagrange Multiplier (DLM) technique^62^ that was originally developed to study particulate flows by Glowinski *et al*.^63^, and Patankar *et al*.^64^. Each particle is explicitly resolved on the Eulerian grid as a separate domain, using solid volume fractions. The fluid equations are solved throughout the entire computational domain; however, Lagrange multiplier constraints are applied inside the particle domain, such that the fluid within any volume associated with a solid particle moves as an incompressible rigid body. The particles interact with the surrounding fluid via fluid dynamics equations, resulting in implicit fluid-rigid-body coupling relations that produce realistic fluid flow around the particles (i.e., no-slip boundary conditions). In addition, particle-particle interactions are implemented using the DEM method developed by Cundall and Strack^65^ with frictional, inelastic contact forces. Since the particle domain is explicitly resolved on the Eulerian grid, we slightly modify this method using the overlap volume of contacting particles as the input to the contact forces. The code is parallelized using the Structured Adaptive Mesh Refinement Application Infrastructure (SAMRAI) framework developed at Lawrence Livermore National Laboratory (LLNL)^66^. This framework allows tracking individual particle position on multiple central processing units (CPUs) and refining the resolution on a structured grid in areas of interest (e.g., solid-fluid interfaces, maximum velocity zones, etc.). Griffith *et al*.^67^,^68^ extended the SAMRAI infrastructure for fluid-structure interaction problems using an immersed boundary method first developed by Peskin *et al*.^69^. Our approach extends this framework using the DLM technique to describe fluid-solid interactions for the case of a large number of rigid particles. Numerical details of the method are described in Kanarska *et al*.^62^.

The code has mainly been used to simulate spherical particle motion in a Newtonian fluid^62^. A few significant modifications in the code were performed to simulate a liquid polymer resin with a dispersion of carbon fibers such as: (a) implicit discretization of the viscosity terms, (b) implementation of variable viscosity that depends on the shear rate,(c) implementation of the second order pressure projection method to include pressure into calculation of the viscosity terms and (d) contact forces were extended to include anisotropic fiber geometry. All of these changes allow efficient simulation of the fiber flow in the nozzle area.

### Global Flow Modeling

The bulk flow within the entire print nozzle assembly was simulated using commercially available CFD software (StarCCM+) to identify potential locations for fiber aggregation and nozzle clogging. The ink was simulated as a homogenous fluid with a non-Newtonian viscosity profile. The ink’s shear thinning behavior was modeled using a power law fit to rheology data obtained via the method previously mentioned. Thus, the local viscosity varies with the local shear rate magnitude according to Eq. 2.


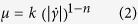


where k and n are the power law consistency index and power law index, respectively and 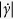 is the magnitude of the local shear rate tensor. A least-square fit of the rheology measurements yielded values of 2.033e3 Pa-s^n^ and 1.775e-1 for k and n, respectively, for the ink at 20 °C. The fluid was given a density of 1120 kg/m^3^.

The computational domain consisted of the internal lumen of the syringe/tip assembly obtained using 3D CAD of the individual components. The domain was discretized using an unstructured, hexahedral cell mesh comprised of a Cartesian core mesh and multiple layers of prismatic cells extruded from the syringe/nozzle wall. In these simulations, an axisymmetry assumption is used to reduce the computational domain size. A no-slip boundary condition is prescribed on the syringe and tip wall. At the inlet of the computational domain, a uniform velocity profile is applied. This average inlet velocity is calculated to yield a flow rate that results in an average velocity at the tip outlet that matches the print head speed (either 0.005 m/s or 0.015 m/s). Given an inlet diameter of 1.58e-2 m, the average inlet velocities used are 1.26e-6 m/s and 3.77e-6 m/s. The steady-state solution of the flow within the syringe/tip assembly is obtained by solving the conservation of mass and momentum equations on the computational mesh.

### Rheological Characterization

Rheological properties of the ink were characterized using a stress-controlled Rheometer (AR 2000ex, TA Instruments) with a cone and plate geometry having a diameter of 40 mm and a cone angle of 2.006°. For fiber filled systems, a cup and bob geometry was used to measure viscosity. Temperature was held at 30 °C via Peltier plate temperature control for both geometries used. A flow sweep from 10^−2^ to 10^1^ Pa at a constant frequency of 1 Hz was conducted to record the viscosity at increasing shear rates.

### Optical Microscopy

A face centered cubic structure with fifty percent void volume was embedded in an epoxy syatem (Struers, EpoFix epoxy kit) and cured overnight. The embedded structure was sectioned using a Buehler, Isomet 1000, slow speed cut off saw. Sections were taken perpendicular and parallel to the prints to visualize the fiber orientation of the printed filaments and were polished manually. Images were taken using Keyence, Model 5000 Digital Microscope.

### Dielectric relaxometry

All Dielectric relaxometry analyses were carried out using a Novocontrol Concept 40 Dielectric Spectrometer system based upon an Alpha-A combined frequency response analyzer/signal generator/charge amplifier assembly and a Quatro cryostat sample temperature control system. The instrument had a working frequency range of 10^−5^ to 10^7^ Hz and a stable sample temperature range of −160 to 300 °C. All BDS analyses reported in this paper were carried out using a computer controlled frequency domain method as described by D. Hayward *et al*.^70^. Samples of ~2 mm thickness and 20 mm in diameter were analyzed under a constant flow of N_2_ in a parallel plate configuration cell with an upper electrode diameter of 10 mm. Liquid resin samples were loaded between both the upper and lower electrode and the gap adjusted by means of micrometer to 2.0 mm. The samples were brought to a constant temperature of 80 ± 0.1 °C in the cryostat and a series of single frequency measurements at 100 Hz and a sample rate of 30 seconds were collected for a total run time of 24 hours. Data were plotted as the imaginary permittivity component (ɛ”) of the complex permittivity as a function of time (where 1/ɛ” is proportional to increased extent of network conversion) in order to assess the extent of cure.

### Mechanical Analysis

Type five dog bones were bones were cut parallel to the raster printing of the filament of the resin and also on random cast sheets according to ASTM D638-10 specifications using a CO_2_ laser cutter. Samples were held via pneumatic grips that were strained to failure at a rate of 1 mm per minute using an Instron 5967 equipped with a 2 kN load cell in ambient temperature. Measurements of thickness and width were taken to calculate the cross-sectional area of the specimens. Specimens having (6 mm × 6 mm × 2.3 mm) were CO_2_ laser cut for compression tests on samples that were raster printed to be parallel to one axis. Samples were compressed parallel and perpendicular to the printed filament and on one corresponding face for the random cast specimens. All samples were measured at ambient temperature at a strain rate of 0.375 mm/min to thirty percent compression using an Instron Electro-Mechanical Test Machine, (Instron 5800R-4505) equipped with a 100 kN load cell. The Young’s modulus was calculated based off of the cross sectional area and force in the linear region of the resultant stress strain curves obtained from tensile testing.

### Electrical Conductivity Characterization

Electrical conductivity was measured on a Keithley 2400 Source meter using the four-probe method described here^71^. Metal electrodes were attached to opposite edges of a rectangular sample, consisting of a single solid layer of AMCFRC. The AMCFRC sample was printed such that the carbon fibers were aligned parallel to the long edge of the rectangular sample. The amount of current transmitted through the sample during measurement was 10 μA and the voltage drop parallel to the current path in the sample was measured over distances of 8–15 mm. Seven or more measurements were taken on each sample. For conductivity measurements parallel to the fibers, current was transmitted through the short edge of the sample. For conductivity measurements perpendicular to the fibers, current was transmitted through the long edge of the sample. Two-probe measurements were also taken to confirm the trends.

## Additional Information

**How to cite this article:** Lewicki, J. P. *et al*. 3D-Printing of Meso-structurally Ordered Carbon Fiber/Polymer Composites with Unprecedented Orthotropic Physical Properties. *Sci. Rep.* **7**, 43401; doi: 10.1038/srep43401 (2017).

**Publisher's note:** Springer Nature remains neutral with regard to jurisdictional claims in published maps and institutional affiliations.

## Supplementary Material

Video S1

Video S2

Supplementary Information
