# Supplementary material for: 3D-Printing of Meso-structurally Ordered Carbon Fiber/Polymer Composites with Unprecedented Orthotropic Physical Properties
Source: Sci Rep. 2017 Mar 6;7:43401. doi: 10.1038/srep43401 (PMC5338294; doi:10.1038/srep43401)
Supplement: Supplementary Information [file srep43401-s3.doc]

Supplementary Information

3D-Printing of Meso-structurally Ordered Carbon Fiber/Polymer Composites with Unprecedented Orthotropic Physical Properties

James P. Lewicki^*^, Jennifer N. Rodriguez, Cheng Zhu, Marcus A. Worsley, Amanda S. Wu, Yuliya Kanarska, John D. Horn, Eric B. Duoss, Jason M. Ortega, William Elmer, Ryan Hensleigh Ryan A. Fellini, and Michael J. King

**
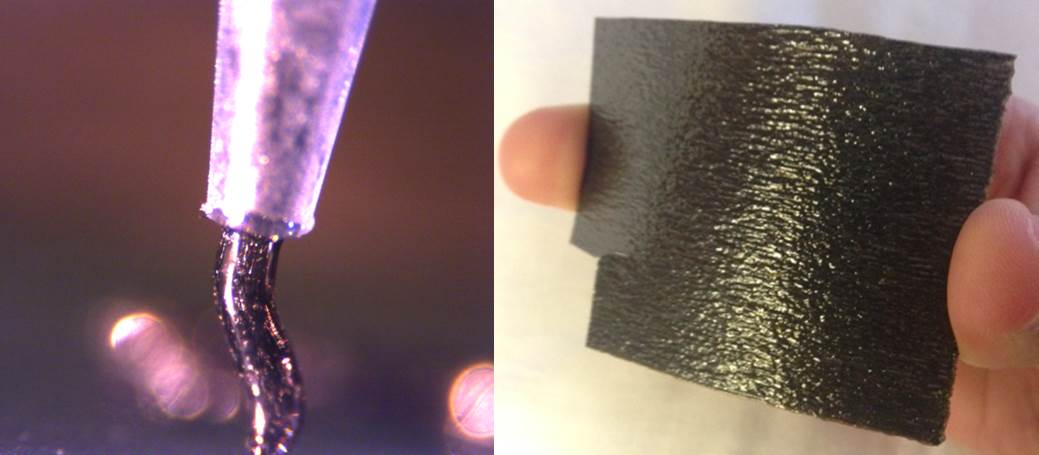
**

**Figure S1**. Left: Extrusion of a 3 volume % AMCFRC Ink through a 250um DIW nozzle. Right: unidirectional printed part (2 layers), printed a 250um filamentary resolution using a 3 vol. % AMCFRC ink.


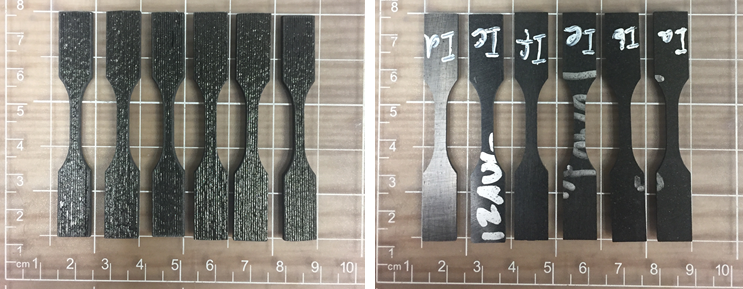


**Figure S2**. Left: 6 Replicate printed and cured AMCFRC in dogbone form for mechanical testing, prior to polishing. Right: 6 Replicate lapped and polished AMCFRC dogbones prior to tensile testing to failure.

**Video S1**. Real time DIW printing of a 8 volume % AMCFRC ink using a 610um exit diameter straight tip

**Video S2**. Thermal snap curing of the homopolymerized (unfilled) LLNL aromatic epoxy resin system. Note that the glass slide is initially at ~ 200 degrees Celsius as measured by a contact thermocouple. When a drop of resin is added the temperature of the resin is observed to climb to ~200 degrees Celsius from room temperature within 10 seconds and upon reaching 180°C then cures in less than 2 seconds thereafter.

**Additional information on alternative nozzle geometries**

The efficiency of the alignment process and the ultimate volume fraction obtainable within a fluid regime for a fiber filled resin is in no small part controlled by and dependent on the geometry of the micro-extrusion nozzle as well as the pre-nozzle fluid reservoir. Many factors including nozzle length, pitch, surface area and wall surface energy for a given fiber volume fraction and aspect ratio may be expected to contribute to the ultimate degree of alignment and achievable volume fraction at a given driving pressure. The authors of this manuscript are currently undertaking a computational design approach to determine optimal geometries for various print conditions and this work will be published separately in 2017, however for illustration purposes here we show in **Figure S2** the clear effects of changing nozzle parameters on the viscosity profile of our resin system.


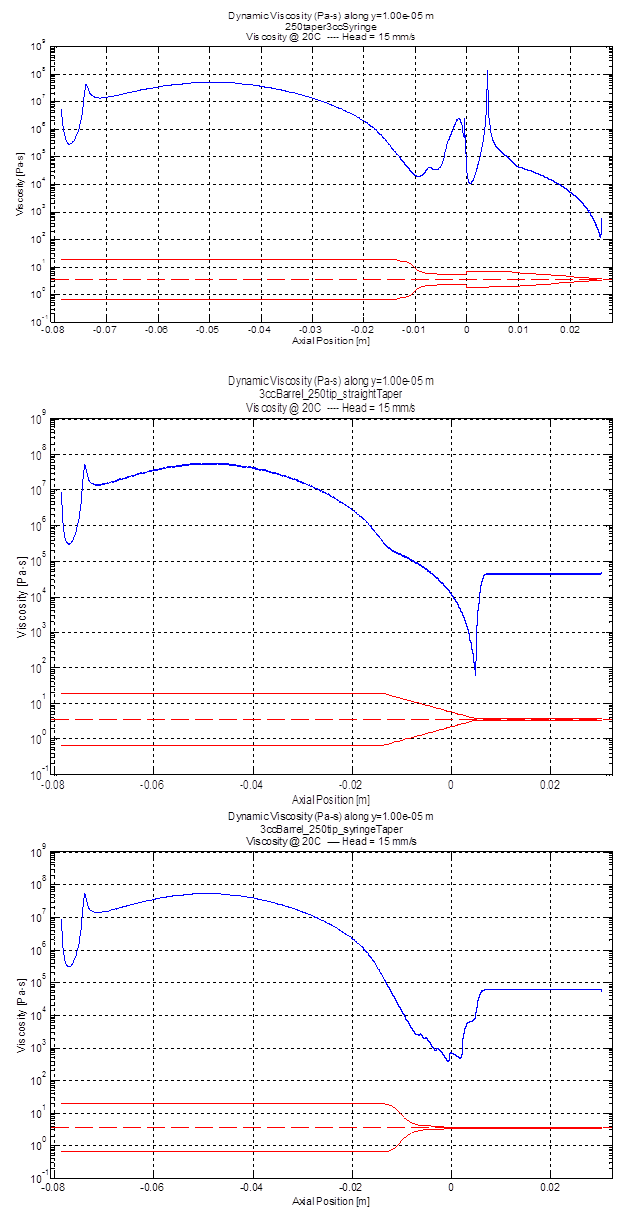


**Figure S2**. Computational fluid dynamics simulations of a continuum fluid approximation of the CF filled resin used in the work reported here. Each simulation predicts the fluid viscosity profile as a function of the axial position along the complete length of a series of DIW fluid delivery systems to the exit of the extrusion nozzle, simulated at a constant driving pressure and at 20°C. Top: volumetric profile of an actual Nordsen luer lock 250 µm tip and syringe barrel delivery system, of the type used for the printing of 3 volume % CF resins at 250 µm feature resolution. Note that there are discontinuous changes in the volume of the barrel and tip system as a consequence of the Luer lock arrangement and these translate to large and abrupt fluctuations in the fluid viscosity within the print head. Such instabilities in viscosity profile are though to negatively impact the stability and alignment of the fiber filled print. Middle and lower images: theoretical modified geometries which eliminate abrupt changes in volume as a function of axial position. Note here that the viscosity profiles may be effectively stabilized by the choice of a different geometry however the optimal geometric solution which yields effective alignment, flow and minimizes blocking is not necessarily simple has is the subject of a current computational design study by the authors.

Furthermore, a detailed computational investigation of the effects of nozzle geometry on fiber alignment over a range of volume fractions has recently been completed by the authors^[1]^ and this work will also be published in 2017

[1] Y. Kanarska, J. P. Lewicki, J. N. Rodriguez, A. Wu, E. B. Duoss, Computers & Fluids Submitted
